# Cerebral venous thrombosis (CVT) complicating tubercular meningitis

**DOI:** 10.1186/s12883-023-03286-4

**Published:** 2023-06-24

**Authors:** Qian Li, Junfeng Han, Yiyi Wang, Yijun Song

**Affiliations:** 1 Department of Neurology, Tianjin Medical University General Hospital, Tianjin, 300052 China; 2 Department of Neurology, Haihe Clinical College of Tianjin Medical University, Tianjin, 300350 China; 3 Department of Tuberculosis, Haihe Hospital, Tianjin University, Tianjin, 300350 China; 4 Department of Neurology, Haihe Hospital, Tianjin University, Tianjin, 300350 China; 5 Department of General Medicine, Tianjin Medical University General Hospital, Tianjin, 300052 China

**Keywords:** Tubercular meningitis, Cerebral venous thrombosis, Magnetic resonance imaging, Venous stroke, Cerebrospinal fluid, Anti-tuberculosis therapy

## Abstract

**Background:**

Tuberculous meningitis (TBM), complicated with cerebral venous thrombosis (CVT), has been sparsely reported and needs to be investigated further.

**Methods:**

Among those with tuberculous meningitis in Haihe Hospital, Tianjin University, 3 patients with venous sinus thrombosis were identified retrospectively. “Tuberculous meningitis” and “cerebral venous thrombosis” were used as keywords, and the retrieved literature was summarized and analyzed. Our data were combined with previously reported case data to describe this new condition.

**Results:**

Among 28 patients with a median onset age of 31 years for TBM, 17 were females. The manifestations were fever, headache, and seizure. Magnetic resonance imaging (MRI) venography showed that the most common site of venous sinus thrombosis involved superior sagittal sinus, left transverse sinus, left sigmoid sinus, cavernous sinus, and straight sinus. The abnormalities found on MRI include hydrocephalus, exudates, hemorrhage, meningeal enhancement, infarction, and tuberculoma. In the acute phase, all patients received standard anti-TB treatment, and 14/28 patients received anticoagulant treatment. The mortality rate of these patients was 17.9%, and 21/28 (75%) became functionally independent.

**Conclusions:**

CVT is one of the rare complications of TMB and must be considered a differential diagnosis in patients with TBM who show poor clinical features and/or develop new neurological signs.

## Background

Central nervous system tuberculosis (CNS TB) is a major cause of morbidity and mortality in developing countries and may present as tubercular meningitis (TBM), meningoencephalitis, tuberculomas, and abscesses. TBM manifests as critical and serious complications. Cerebral venous thrombosis (CVT) is characterized by a thrombus in the dural venous sinuses. The association of TBM with CVT is rare. However, most of the previous studies were case reports. Herein, we analyzed the clinical and radiological features, treatment responses, and outcomes of the patients with tuberculous meningitis (TBM) complicated with cerebral venous thrombosis (CVT). Subsequently, we combined our data with those reported previously to outline the features of this rare co-morbidity.

## Methods

### Current case series

This study was approved by the Ethics Committee of Haihe Hospital, Tianjin University. Among the patients with tuberculous meningitis hospitalized from January 2018–May 2021, 3 were complicated with venous sinus thrombosis. These 3 patients were diagnosed with tuberculous meningitis according to the consensus scoring system of The Lancet [evidence of positive culture for *Mycobacterium tuberculosis* in the CSF(cerebrospinal fluid) samples). All 3 patients underwent head MRV( magnetic resonance venography) examination, which indicated clear diagnosis of venous sinus thrombosis. All patients were followed up and had good prognosis.

### Literature review

To further clarify the clinical features of patients with TBM and cerebral sinus venous thrombosis, we searched PubMed (Medline) through January 14, 2022, for articles published in English using the search string (“cerebral venous and sinus thrombosis, cerebral venous thrombosis” [MeSH Terms] OR “cortical vein thrombosis” OR “intracranial thrombosis”) AND (“Tubercular Meningitis ” [MeSH Terms]). We also searched the references for related published articles. The retrieved articles were reviewed to identify cases with TBM and cerebral sinus venous thrombosis.

After combining our data with those from previously reported cases, the clinical and radiological features, treatment, and outcomes of the patients with TBM and cerebral sinus venous thrombosis were analyzed further to characterize the new condition.

## Results

### Results from the present case series study

#### Case #1

**Fig. 1:**
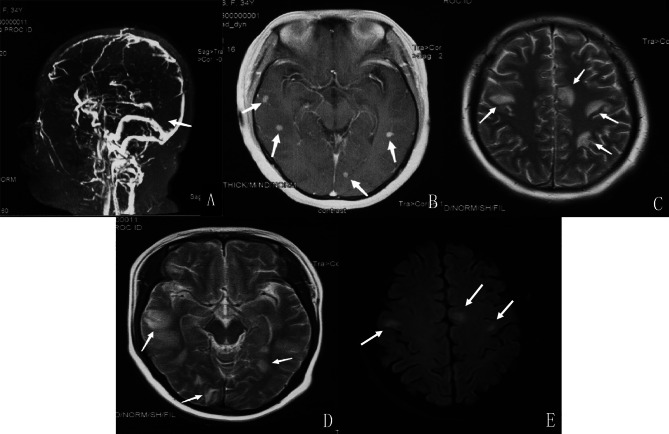
Radiographic findings of the patient in case 1. **A:** MRV shows partial visualization with thrombosis of the straight sinus; **B:** T1-weighted MRI of the brain with contrast images showing enhancement of multiple nodules in the temporal and occipital lobes; **C**, **D**, and **E:** MRI revealed infarction of bilateral frontoparietal temporal and left occipital lobes

A 34-year-old female was admitted because of fever and headache. She got pregnant with assisted reproductive technology 6 months ago and delivered a dead baby under the protection of the perineum 8 days before admission. The general physical examination was unremarkable, and the neurological examination was normal. CSF test showed that gene X-pert was positive for *M. tuberculosis*, which was confirmed by metagenomics next-generation sequencing (mNGS). Brain magnetic resonance imaging (MRI) of the brain showed an abnormal signal shadow of the bilateral parietal lobe. MRI venography showed partial visualization with thrombosis of the straight sinus, suggesting CVT (Fig. 1). She was treated with anti-tuberculosis drugs and low-molecular-weight heparin. The patient was treated in the hospital for 2 months and continued to take oral warfarin for 6 months, and ingested anti-TB drugs regularly for 14 months. After completing the regimen, the patient was followed up for 3 months; the last modified Rankin score (mRS) of the patient was 0.

#### Case #2

**Fig. 2:**
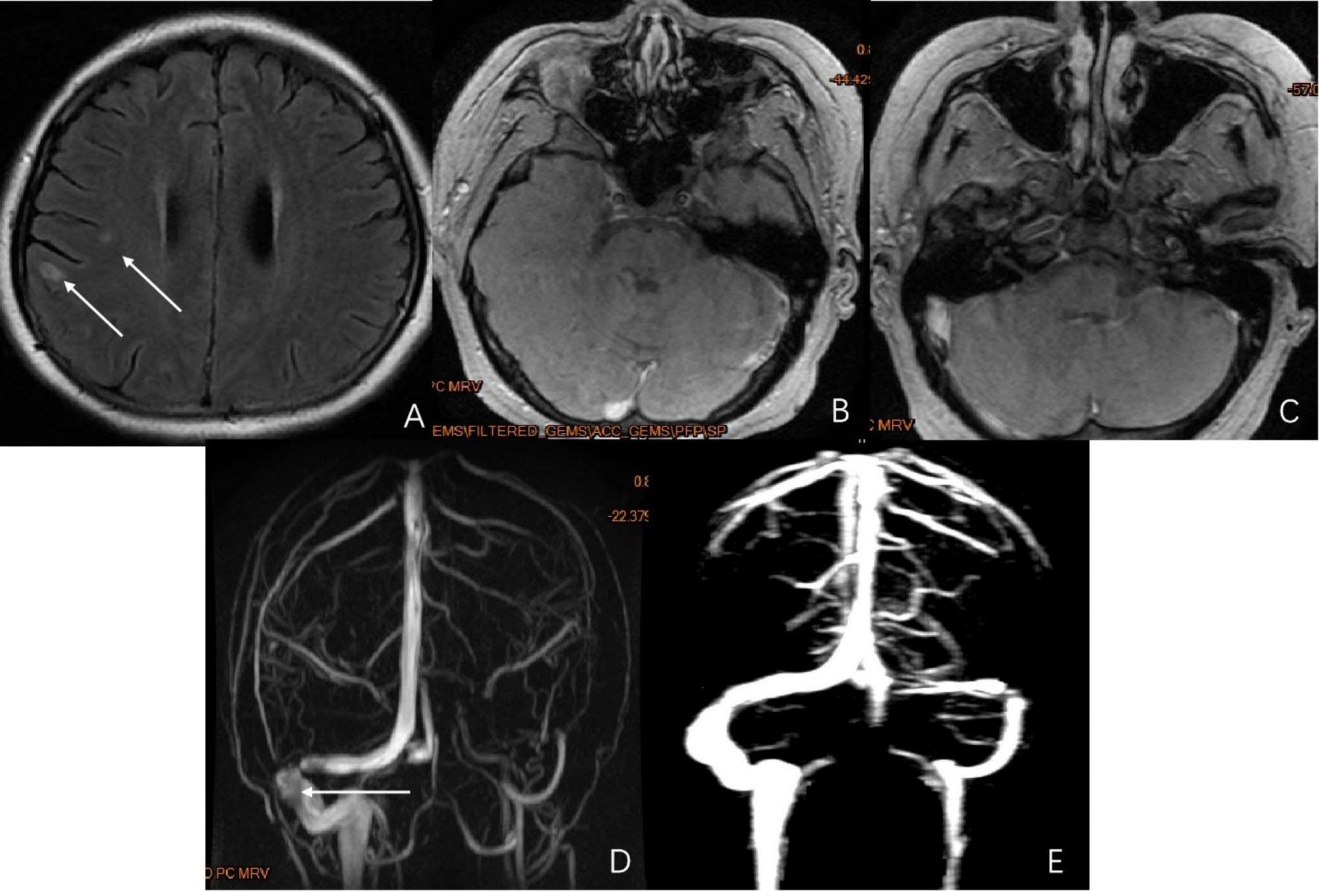
Radiographic findings of the patient in case 2. **A:** Brain plain MRI FLAIR sequence shows multiple high-signal intensity patches in bilateral frontal parietal cortex and subcortical white matter. **B:** MRV shows a locally narrowed right sigmoid sinus, while the left transverse sinus and sigmoid sinus are not shown. **C** and **D:** Post-contrast MRI T1WI shows filling defects in the right sigmoid sinus, left transverse sinus, and sigmoid sinus. **E:** After 6 months, the re-examination image shows that the right sigmoid sinus is normal, and the filling defect of the left transverse sinus and sigmoid sinus was better than before

A 29-year-old female was admitted with fever, headache, vomiting, and diplopia. She had a history of induced abortion 2 months before admission. mNGS detected *M*. tuberculosis in CSF samples. MRI of the brain showed a focal infection in the left temporoparietal lobe with enhanced manifestations. MRI venography showed thrombosis of the left transverse sinus and sigmoid sinus (Fig. 2). She received anti-TB drugs and low molecular weight heparin for 2 months. After discharge, she continued to take rivalsaban orally for 6 months and anti-TB drugs regularly for 14 months. After completion of the medication, the patient was followed up for 3 months, and her last mRS was 0.

#### Case #3

**Fig. 3:**
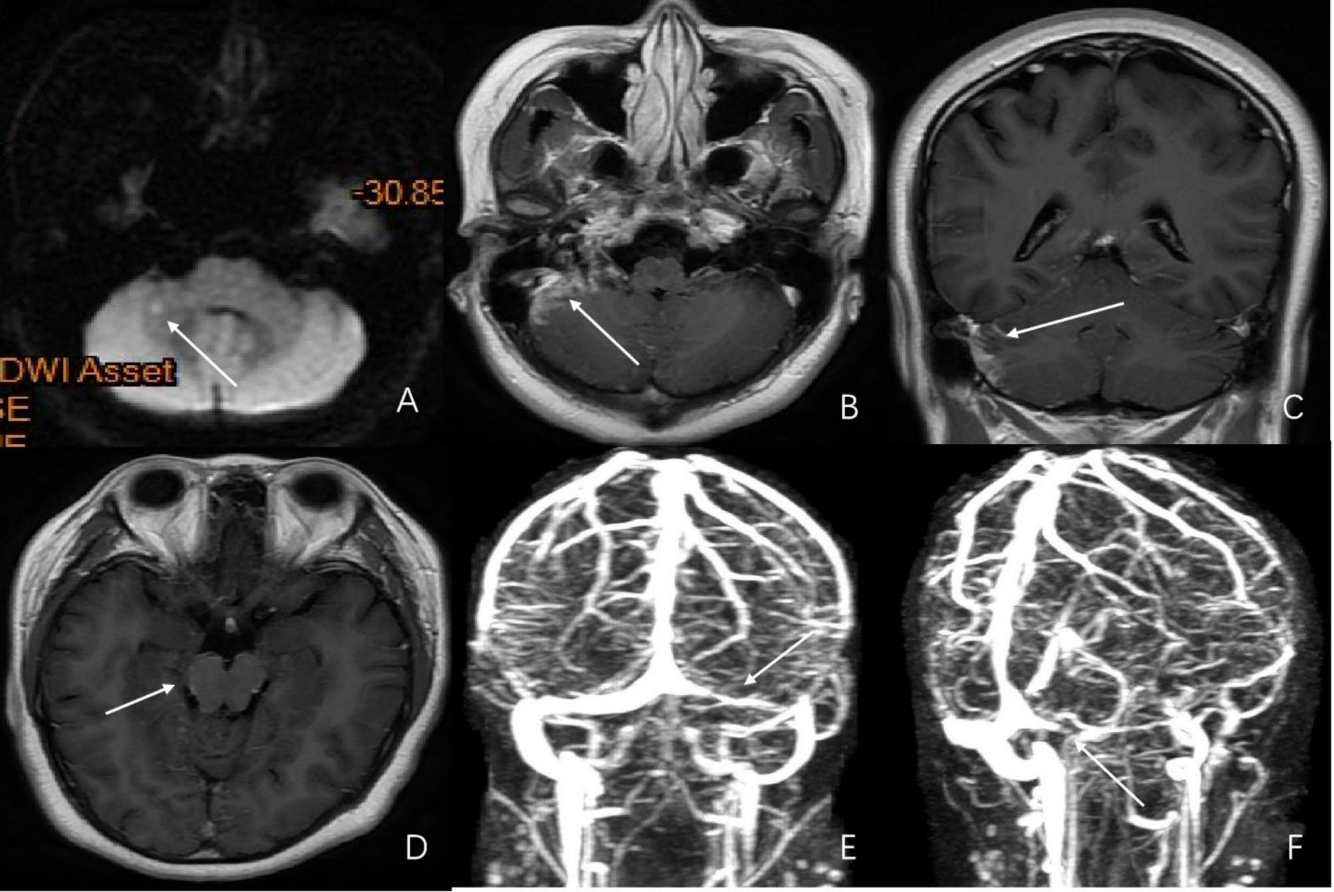
Radiographic findings of the patient in case 3. **A:** Brain plain MRI DWI sequence shows high-signal intensity nodules in the right cerebellum. **B**, **C**, and **D:** Post-contrast MRI T1WI (axial and coronal) shows patchy enhancement around the right cerebellum near the right sigmoid sinus area and enhancement of pia mater of the basal cistern around the midbrain. **E** and **F:** Post-contrast MRI T1WI shows filling defects in the left transverse sinus and sigmoid sinus

A 30-year-old female was admitted with fever, headache, and vomiting. She was healthy in the past. The general physical examination and the nervous system were normal. The mNGS of CSF showed the presence of *M*. tuberculosis. Brain MRI indicated multiple tuberculomas in the brain. MRI venography showed thrombosis of the left transverse sinus and sigmoid sinus (Fig. 3). She was treated with anti-TB drugs and low-molecular-weight heparin for 2 months. After discharge, she continued oral administration of anti-TB drugs. The prognosis was satisfactory, and the patient was followed up for 6 months after discharge; the last mRS was 0.

The characteristics of the patients are summarized in Table 1.

**Table 1:** Summary of clinical characteristics of the patients

Item	Case 1	Case 2	Case 3
Sex	female	female	female
Age (years)	34	29	30
Pulmonary tuberculosis	Yes	Yes	No
Other risk factors	Assisted reproductive technology; artificial abortion	Artificial abortion	No
Clinical manifestation	Fever, headache, paralysis of the right upper limb	Fever, headache, vomiting, diplopia	Fever, headache, vomiting
MRV finding	Partial visualization with thrombosis of the straight sinus	Thrombosis of the left transverse sinus and sigmoid sinus	Thrombosis of the left transverse sinus and sigmoid sinus
MRI finding	Infarction	Focal infection	Tuberculomas
Treatment	Anti-tubercular therapy (isoniazid, rifampicin, pyrazinamide, ethambutol, and moxifloxacin)	Anti-tubercular therapy (isoniazid, rifampicin, pyrazinamide, ethambutol, and linezolid)	Anti-tubercular therapy (isoniazid, rifampicin, pyrazinamide, and ethambutol)
	Hydrocortisone and mannitol	Methylprednisolone and mannitol	Hydrocortisone and mannitol
	Anticoagulants (low molecular weight heparin once a day, 2 months; oral warfarin, 6 months)	Anticoagulants (low molecular weight heparin once a day, 2 months; oral rivarsaban, 6 months)	Anticoagulants (low molecular weight heparin once a day, 2 months)
Outcome	Last mRS 0	Last mRS 0	Last mRS 0

### Results from a literature review and pooled data analysis

The initial literature search yielded 11 published reports. The reports and the references were reviewed to identify any related published articles. A total of 25 patients with TBM and cerebral sinus venous thrombosis were identified. After pooling the data, the characteristics of the previously reported and our 3 patients are summarized (Table 2).

**Table 2:** Characteristics of 28 patients with tuberculous meningitis and cerebral sinus venous thrombosis

Characteristics	(n = 28)
Age at onset, median (range), y	31 (1–61)
Sex (female), n (%)	17 (60.7%)
Pediatric,n(%)	5 (17.9%)
Pulmonary tuberculosis, n (%)	6 (21.4%)
Other risk factors	
Spondylitis with epidural abscess	1 (3.6%)
Use of oral contraceptives	1 (3.6%)
Artificial pregnancy	1 (3.6%)
Artificial abortion	2 (7.1%)
Clinical manifestation, n (%)	
Fever	24 (85.7%)
Headache	23 (82.1%)
Seizure	18 (64.3%)
Limb paralysis	10 (35.7%)
Disturbance of consciousness	5 (17.9%)
Cranial nerve paralysis	5 (17.9%)
Vomiting	7 (25.0%)
Paresthesia	3 (10.7%)
Diplopia	3 (10.7%)
Visual obscuration	2 (7.1%)
MRV finding, n (%)	
Superior sagittal sinus	17 (60.7%)
Left transverse sinus	11 (39.3%)
Left sigmoid sinus	9 (32.1%)
Straight sinus	2 (7.1%)
Cavernous sinus	2 (7.1%)
Right transverse sinus	1 (3.6%)
Right sigmoid sinus	0 (0.0%)
MRI finding, n (%)	
Hydrocephalus	12 (42.9%)
Exudates	11(39.3%)
Hemorrhage	6 (21.4%)
Meningeal enhancement	6 (21.4%)
Infarction	4 (14.3%)
Tuberculoma	4 (14.3%)
Treatment	
Anti-tubercular therapy	28 (100.0%)
Anticoagulants	14 (50.0%)
Heparin	7 (25.0%)
Oral warfarin	4 (14.3%)
Rivaroxaban	1 (3.6%)
Unknown	5 (17.9%)
Vitamin K antagonist	2 (7.1%)
Aspirin	1 (3.6%)
Surgery	1 (3.6%)
Outcome, n (%)	
Last mRS (0–2)	21 (75.0%)
Last mRS (3–5)	2 (7.1%)
Last mRS (6)	5 (17.9%)

The median onset age of the patients was 31 (range: 1–61) years, and 17 were females. The manifestations were mainly fever (85.7%), headache (82.1%), and seizure (64.3%). According to the magnetic resonance imaging venography (MRV) findings, the most common sites of venous sinus thrombosis were superior sagittal sinus (60.7%), left transverse sinus (39.3%), and left sigmoid sinus (32.1%). The abnormalities detected on MRI were hydrocephalus (42.9%), exudates (39.3%), hemorrhage (21.4%), meningeal enhancement (21.4%), infarction (14.3%), and tuberculoma (14.3%). In the acute phase, all patients received standard anti-TB treatment, while 14/28 (50.0%) of patients received anticoagulant treatment, and 1/28 received oral aspirin. The mortality rate of these patients was 17.9%. Subsequently, 21/28 (75%) became functionally independent [the last modified Rankin scale (mRS) ≤ 2].

## Discussion

Venous sinus or cerebral veins is a specific anatomical location of venous thrombosis [1] and a particular type of stroke, which commonly affects young adults, with 75% of events occurring in women. The clinical manifestations of CVT are highly variable. The symptoms and signs of CVT can be grouped into presenting syndromes; the most frequent types are isolated intracranial hypertension syndrome (manifesting as headache, vomiting, papilledema, or visual problems), focal neurological deficits (hemiparesis, dysphasia, cranial nerve deficits, and altered sensorium), and encephalopathy (with multifocal signs and altered mental status [2]. The less frequent presentation is cavernous sinus syndrome or syndromes of multiple palsies of the lower cranial nerves. The risk factors associated with CVT include oral contraceptives, pregnancy, head injury, neurological procedures, lumbar puncture, infections, otitis and mastoiditis, and meningitis [2]. Typically, these risk factors are associated with the thrombogenic triad of Virchow, including vessel wall injury, blood stasis, and hypercoagulability [3].

Vascular endothelial injury caused by infections (bacterial, TB, fungal), especially meningitis, is one of the risk factors of CVT [4], and a few studies have reported TBM complicated with CVT; however, its true prevalence is uncertain. Tuberculous meningitis often occurs in an environment with insufficient resources. Also, since tuberculous meningitis and CVT have the same clinical manifestations (such as headache, epilepsy, and limb paralysis caused by cerebral infarction), not all TBM patients undergo MRI or CT venous examination. Low detection rates are likely to contribute to low reported prevalence rates of this complication. In the current study, TBM was the main factor of CVT in these patients [5–12]. In addition, one patient was complicated with spondylitis with epidural abscess, one patient used oral contraceptives [13], and one case was combined with coronavirus disease-19 (COVID-19) [14]. Herein, 2/3 patients were sick after artificial abortion, and one was pregnant by assisted reproductive technology.

The current study demonstrated diverse and nonspecific clinical manifestations of TBM patients complicated with CVT, such as fever, headache, and epilepsy. Seizures are common manifestations in patients with TBM or CVT, especially in the acute phase of the disease. Previous studies have shown that seizures occur in 34% of patients with TBM, and the incidence of seizures in CVT patients is 44.3% [15]. In this study, adult cases had a high incidence of epilepsy (64.3%), suggesting that when adult patients with TBM have seizures, we should be alert to whether the patients also present CVT concurrently.

The most common sites of CVT complicated with TBM were superior sagittal sinus (65.4%), left transverse sinus (34.6%), and left sigmoid sinus (26.9%). These findings were similar to those of International Study of Cerebral Venous and dural sinus Thrombosis (ISCVT), indicating that the most common sites of CVT are superior sagittal (62%) and left lateral sinuses (44.7%), including transverse and sigmoid sinuses [2].

Next, we analyzed the possible mechanisms of TBM complicated with CVT, including endothelial injury due to inflammatory response, sluggish venous flow, increased platelet aggregation, and release of pro-coagulant factors [16]. The host inflammatory response is critical in TBM pathology [17]. Microglial cells and migrated infected neutrophils and macrophages are rapidly activated and secrete cytokines and chemokines [18], such as tumor necrosis factor-alpha (TNF-a), interleukin-6 (IL-6), IL-1b, CCL2, CCL5, and CXCL10 [19]. TNF-a and IL-6 have an additive effect in pro-coagulant activity on human endothelial cells via platelet aggregation and thrombosis [20]. The other host mediators implicated in the pathology of TBM include matrix metalloproteinases (MMPs) and vascular endothelial growth factor (VEGF). Moreover, VEGF is a potent factor in vascular permeability and angiogenesis [20]. It is also vasculotoxic, prothrombotic, reduces cerebral blood flow, and produces nitric oxide and oxygen free radicals [21]. In our cases, two patients had a history of induced abortion, and one had undergone artificial assisted pregnancy, which is a high-risk factor for CVT.

Taken together, anti-TB treatment is essential. In the cases summarized above, all patients received standardized anti-TB treatment for TBM; anticoagulation therapy is the mainstay of CVT treatment. The currently recommended treatment is anticoagulation with heparin, followed by oral anticoagulation for 3–6 months. In the current study, 50% of patients received anticoagulant therapy for CVT. Two other patients received other treatment (one child used aspirin; another patient received emergency surgery because of critical illness and died shortly after surgery). The other 12 patients did not receive anticoagulation treatment due to safety concerns as they believed that anticoagulants in infectious venous thrombosis may lead to new cerebral hemorrhage. Our study concluded that most patients who received anticoagulation therapy had a good prognosis (last mRS < 2), and no new cerebral hemorrhage was indicated. Of the 7 patients with poor prognosis (including 2 with last mRS 3–5 and 5 with last mRS 6), only 1 received anticoagulant treatment. This finding suggested that anticoagulant therapy improves the prognosis of patients.

## Conclusion

CVT is one of the rare complications of TBM. Venous stroke may contribute to unexplained worsening of the neurological status. Thus, CVT must be sought as a differential diagnosis in patients with TBM showing worsening clinical features and/or new neurological signs.

## Data Availability

All data generated or analyzed during this study are included in this published article.

## Abbreviations

TBM: tuberculous meningitis
CVT: cerebral venous thrombosis
MRI: magnetic resonance imaging
CNS TB: central nervous system tuberculosis
mNGS: metagenomics next-generation sequencing
CSF: cerebrospinal fluid
COVID-19: coronavirus disease-19
ISCVT: International Study of Cerebral Venous and dural sinus Thrombosis
TNF-a: tumor necrosis factor-alpha
IL-6: interleukin-6
MMPs: matrix metalloproteinases
VEGF: vascular endothelial growth factor
mRS: modified Rankin scale
